# Post-paralysis tyrosine kinase inhibition with masitinib abrogates neuroinflammation and slows disease progression in inherited amyotrophic lateral sclerosis

**DOI:** 10.1186/s12974-016-0620-9

**Published:** 2016-07-11

**Authors:** Emiliano Trias, Sofía Ibarburu, Romina Barreto-Núñez, Joël Babdor, Thiago T. Maciel, Matthias Guillo, Laurent Gros, Patrice Dubreuil, Pablo Díaz-Amarilla, Patricia Cassina, Laura Martínez-Palma, Ivan C. Moura, Joseph S. Beckman, Olivier Hermine, Luis Barbeito

**Affiliations:** Institut Pasteur de Montevideo, Mataojo 2020, Montevideo, 11.400 Uruguay; Imagine Institute, Hôpital Necker, 24 boulevard du Montparnasse, 75015 Paris, France; INSERM UMR 1163, Laboratory of Cellular and Molecular Mechanisms of Hematological Disorders and Therapeutic Implications, Paris, France; Paris Descartes–Sorbonne Paris Cité University, Imagine Institute, Paris, France; CNRS ERL 8254, Paris, France; Laboratory of Excellence GR-Ex, Paris, France; Equipe Labélisée par la Ligue Nationale contre le cancer, Paris, Cedex France; AB Science, 3 Avenue Georges V, 75008 Paris, France; CRCM, [Signaling, Hematopoiesis and Mechanism of Oncogenesis], Inserm, U1068, Institut Paoli-Calmettes, Aix-Marseille Univ, UM105, CNRS, UMR7258, Marseille, F-13009 France; Laboratorio de Neurobiología Celular y Molecular, Instituto de Investigaciones Biológicas Clemente Estable, Montevideo, Uruguay; Departamento de Histología y Embriología, Facultad de Medicina, Universidad de la República, Montevideo, Uruguay; Linus Pauling Institute, Department of Biochemistry and Biophysics, Environmental Health Sciences Center, Oregon State University, Corvallis, USA; Department of Hematology, Necker Hospital, Paris, France; Centre national de référence des mastocytoses (CEREMAST), Paris, France

**Keywords:** ALS, Aberrant glial cells, Neurodegeneration, Masitinib, M-CSF

## Abstract

**Background:**

In the SOD1^G93A^ mutant rat model of amyotrophic lateral sclerosis (ALS), neuronal death and rapid paralysis progression are associated with the emergence of activated aberrant glial cells that proliferate in the degenerating spinal cord. Whether pharmacological downregulation of such aberrant glial cells will decrease motor neuron death and prolong survival is unknown. We hypothesized that proliferation of aberrant glial cells is dependent on kinase receptor activation, and therefore, the tyrosine kinase inhibitor masitinib (AB1010) could potentially control neuroinflammation in the rat model of ALS.

**Methods:**

The cellular effects of pharmacological inhibition of tyrosine kinases with masitinib were analyzed in cell cultures of microglia isolated from aged symptomatic SOD1^G93A^ rats. To determine whether masitinib prevented the appearance of aberrant glial cells or modified post-paralysis survival, the drug was orally administered at 30 mg/kg/day starting after paralysis onset.

**Results:**

We found that masitinib selectively inhibited the tyrosine kinase receptor colony-stimulating factor 1R (CSF-1R) at nanomolar concentrations. In microglia cultures from symptomatic SOD1^G93A^ spinal cords, masitinib prevented CSF-induced proliferation, cell migration, and the expression of inflammatory mediators. Oral administration of masitinib to SOD1^G93A^ rats starting after paralysis onset decreased the number of aberrant glial cells, microgliosis, and motor neuron pathology in the degenerating spinal cord, relative to vehicle-treated rats. Masitinib treatment initiated 7 days after paralysis onset prolonged post-paralysis survival by 40 %.

**Conclusions:**

These data show that masitinib is capable of controlling microgliosis and the emergence/expansion of aberrant glial cells, thus providing a strong biological rationale for its use to control neuroinflammation in ALS. Remarkably, masitinib significantly prolonged survival when delivered after paralysis onset, an unprecedented effect in preclinical models of ALS, and therefore appears well-suited for treating ALS.

**Electronic supplementary material:**

The online version of this article (doi:10.1186/s12974-016-0620-9) contains supplementary material, which is available to authorized users.

## Background

Amyotrophic lateral sclerosis (ALS) is a paralytic neurodegenerative disease characterized by the progressive degeneration of upper and lower motor neurons. Survival after diagnosis varies between 1 and 5 years or more, largely determined on the rate of spread of motor neuron pathology. Paralysis progression in rodent models of ALS appears to be modulated by glial cells that proliferate and express inflammatory mediators in the degenerating spinal cord [1–4]. In the SOD1^G93A^ mutant rat model of ALS, a rapid spread of paralysis is associated with marked glial cell activation and the emergence of aberrant glial cells that actively proliferate around degenerating motor neurons [4, 5]. Furthermore, aberrant glial cells display a marked neurotoxic potential on cultured motor neurons [4], suggesting that they might directly contribute to the rapid spread of paralysis of ALS rats. It remains unknown, however, whether pharmacologically downregulation of aberrant glial cells could slow paralysis progression in the rat model of ALS.

We have theorized that aberrant glial cells proliferating in the ALS spinal cord could be sensitive to tyrosine kinase inhibitors that target the family of type III growth factor receptors including PDGF-R, c-Kit, FLT3, and CSF-1R. These receptors synergistically signal cell proliferation and the migration of cancer and hematopoietic cells, including macrophages [6]. In particular, while M-CSF/CSF-1R signaling is critical for the mononuclear phagocytic system [7, 8], a recent report indicates that damaged motor neurons induce the expansion of spinal cord microglia by expressing M-CSF [9]. Thus, we have explored whether the inhibition of CSF-1R and related kinase receptors could modulate neuroinflammation and slow disease spreading in an inherited rat model of ALS.

Among candidate tyrosine kinase inhibitors, masitinib (AB1010) was found to be a highly selective kinase inhibitor [10, 11] and shown to prevent central nervous system (CNS) neuroinflammation in multiple sclerosis [12], stroke [13], and Alzheimer’s disease [14]. Thus, we aimed to characterize the effects of masitinib on cultured aberrant glial cells and determine its therapeutic potential after oral administration to SOD1^G93A^ rats. Because aberrant glial cells emerge only after paralysis onset [4], the drug treatment was initiated following overt disease onset to better simulate the clinical condition of ALS patients. We found that masitinib inhibited glial cell activation in SOD1^G93A^ rats and prolonged survival, indicating a promising therapeutic approach to ALS.

## Methods

### Animals

Male SOD1^G93A^ progeny were used for further breeding to maintain the line. Rats were housed in a centralized animal facility with a 12-h light-dark cycle with ad libitum access to food and water. Perfusion with fixatives was performed under 90 % ketamine—10 % xylazine anesthesia and all efforts were made to minimize animal suffering, discomfort, or stress. All procedures using laboratory animals were performed in accordance with the national and international guidelines and were approved by the Institutional Animal Committee for animal experimentation. Male hemizygous NTac:SD-TgN (SOD1^G93A^)L26H rats (Taconic), originally developed by Howland et al. [15], were bred locally by crossing with wild-type nontransgenic Sprague-Dawley female rats.

### Determination of disease onset and end-stage

All rats were weighed and evaluated for motor activity daily. Disease onset was determined for each animal when pronounced muscle atrophy was accompanied by abnormal gait, typically expressed as subtle limping or dragging of one hind limb. End-stage was defined by a lack of righting reflexes or the inability to reach food and water.

### Masitinib post-paralysis survival trial

Only transgenic rats showing weakness and gait alterations in hind limbs as first clinical sign were selected for masitinib treatment studies. Male and female rats were divided randomly into the masitinib or vehicle-treated groups. Masitinib mesylate (AB1010) freshly prepared in drinking sterilized water was administrated daily at a dose of 30 mg/kg using a curved stainless steel gavage needle with 3-mm ball tip. Dosing was defined in accordance to previous studies in a rat model of stroke that was shown to be safe for chronic treatments [13]. Rats were treated from day 1 or day 7 post-paralysis during 20 days or until end-stage, when they were euthanized.

### Immunohistochemical staining of rat spinal cords

After 20 days of treatment using 30 mg/kg/day of masitinib, starting after paralysis onset, animals were deeply anesthetized and transcardial perfusion was performed with 0.9 % saline and 4 % paraformaldehyde in 0.1 M PBS (pH 7.2–7.4). Fixed spinal cord was removed, post-fixed by immersion for 24 h, and then transverse sectioned (30 μm) in a Leica cryostat. Serial sections were collected in 100 mM PBS for immunohistochemistry. Free-floating sections were permeabilized for 30 min at room temperature with 0.3 % Triton X-100 in PBS, passed through washing buffered solutions, blocked with 5 % BSA:PBS for 1 h at room temperature, and incubated overnight at 4 °C in a solution of 0.3 % Triton X-100 and PBS containing the primary antibodies, rabbit anti-GFAP (1:500, Sigma), mouse anti-S100β (1:400, Sigma), rabbit anti-Iba1 (1:300, abcam), rabbit anti-CD206 (1:300 abcam), mouse anti-CD68 (1:200, abcam), mouse anti-ChAT (choline acetyltransferase) (1:300, Millipore). After washing, sections were incubated in 1:1000-diluted secondary antibodies conjugated to Alexa Fluor 488 and/or Alexa Fluor 633 (1:1000, Invitrogen). Antibodies were detected by confocal microscopy using a confocal LEICA TCS-SP5-DMI6000 or a confocal ZEISS LSM 780.

### Analysis of glial cells in the lumbar spinal cord of hind limb symptomatic rats

The number of aberrant glial cells co-expressing the astrocytic markers GFAP and S100β or the microglia markers Iba-1, CD206, and CD68 were assessed by counting the respective positive cells for the different markers in gray matter from the lumbar cord of SOD1^G93A^ asymptomatic or symptomatic rats that had been treated with either vehicle or masitinib. The analysis was performed manually in at least 20 histological sections per animal (four different rats for each condition) using the cell counter tool of the ImageJ software. For aberrant glial cell counting, values were expressed as the number of GFAP+/S100β+ cells in each ventral horn of the spinal cord in masitinib-treated rats relative to vehicle-treated rats. For microgliosis analysis, the number of Iba1+, CD206+, or CD68+ cells was assessed via manual counting using the ImageJ software tools. Only microglia cells present in the grey matter of the ventral horn of the spinal cord were taken into consideration. Statistical studies were performed using statistical tools of the free Software PAST3. Descriptive statistics were used for each group, and Kruskal-Wallis analysis or one-way ANOVA, followed by Scheffé post hoc comparison if necessary, was used among groups. All results are presented as mean ± SEM, with *p* < 0.01 considered significant.

### Analysis of microgliosis spreading along the degenerating spinal cord of SOD1^G93A^ rats

The spinal cord of hind limb symptomatic rats was dissected at thoracic and cervical levels. Three masitinib-treated rats were compared with three vehicle-treated rats. As previously described, immunohistochemistry was assessed to determine the levels of microgliosis by Iba1 detection. At least ten different 30 μm sections of each rat were visualized using a LEICA TCS-SP5-DMI6000 confocal microscope.

### Analysis of motor neuron number and size

The number of motor neurons expressing ChAT was assessed by counting the positive cells in the gray matter of the lumbar spinal cord of non-transgenic compared with symptomatic SOD1^G93A^, vehicle-, and masitinib-treated rats. Motor neuron counting was based on a stereological approach as previously reported [16]. Briefly, ChAT positive cells were quantified on five 30 μm sections taken 300 μm apart from the ventral horn, comparing the cell numbers in Rexed laminae VII and IX, which display low and high density of large motor neurons, respectively. Results are presented as mean ± SEM, with *p* < 0.01 considered significant. The longest axis (length) of each soma was taken into consideration to quantify the mean size of motor neuron soma. The analysis was performed manually in at least 25 histological sections per animal (four different rats for each condition) using the cell counter tool of the ImageJ software. Results are presented as median ± SD, with *p* < 0.01 considered significant. Statistical studies were performed using statistical tools of the free Software PAST3. Descriptive statistics were used for each group, and Kruskal-Wallis analysis or one-way ANOVA, followed by Scheffé post hoc comparison if necessary, was used among groups.

### Microglia cell cultures from symptomatic SOD1^G93A^ rats

Microglial cells were obtained from a primary culture adult spinal cord of symptomatic SOD1^G93A^ rats according to the procedures described by Trias et al. [5] with minor modifications. Briefly, animals were euthanized by administering an overdose of ketamine/xylazine, and the spinal cord was dissected on ice. After the meninges were removed, the spinal cord was chopped finely and dissociated with 0.25 % trypsin in calcium-free buffer for 5 min at 37 °C. Trypsin treatment was stopped by adding DMEM/10 % (vol/vol) FBS in the presence of 50 μg/mL DNaseI and mechanical disaggregation by repeated pipetting. The resulting extract was passed through an 80-μm mesh to eliminate tissue debris and was then spun. The pellet was resuspended in culture medium [DMEM/10 % (vol/vol) FBS, Hepes (3.6 g/L), penicillin (100 IU/mL), and streptomycin (100 μg/mL)] and was then plated in a 24-multiwell culture dish. Culture medium was removed after 24 h and subsequently replaced every 24 or 48 h depending on the procedure.

### Microglia migratory capacity assay

Primary cultures were plated in high-density 4-multiwell plates during 4 days. After that, and when cells already reached confluence, media was changed to DMEM-0.5 % FBS, thereby, significantly reducing the proliferation rate. A scratch was then made in the monolayer using a 1000 μL tip, and cells were treated with increasing doses of masitinib in DMSO (used as vehicle in control-treated cells). Post-scratch pictures were taken at 24 h using a bright field NIKON microscope attached to a Canon HD camera, and cells that invaded the scratch were counted manually in at least 12 different pictures (three different rats were cultured for this experiment, *n* = 3) using the cell counter tool of ImageJ software.

### Proliferation assay induced by macrophages-colony stimulating factor (M-CSF)

Cells were isolated as described above from the three different symptomatic rat spinal cords and plated in 24-multiwell dishes during 24 h in low serum, DMEM-0.5 % FBS. Cells were then treated with 30 ng/mL of rat M-CSF in PBS-0.1 % BSA (vehicle-treated cells were treated with the same amount of PBS-0.1 % BSA). To determine the inhibitory capacity of masitinib against the tyrosine kinase receptor CSF-1R, cells were treated with increasing doses of the drug (0.01–1 μM) in the presence of M-CSF and compared with vehicle-treated cells for which masitinib was substituted with DMSO. In total, three experimental groups were analyzed: control cells (in DMEM-0.5 % FBS + PBS-0.1 % BSA), vehicle-treated cells (DMEM-0.5 % FBS + M-CSF + DMSO), and masitinib-treated cells (DMEM-0.5 % FBS + M-CSF + masitinib). All wells were treated at the same time with 10 μM of BrdU (Sigma). After 24 h, cells were fixed and immunocytochemistry using anti-BrdU antibody was followed. Briefly, cells were washed and fixed with cold methanol during 5 min at 4 °C, then washed with PBS and treated with 2 M of HCl for 30 min. Cells were blocked using 5 % of BSA in PBS for 1 h, and rat anti-BrdU was incubated for 24 h at 4 °C. After that, the primary antibody was removed, washed with PBS three times for 10 min, and goat anti-rat antibody was incubated for 1 h at room temperature. After washing away the secondary antibody, cells were covered in glycerol mounting medium with 1/2000 DAPI dilution and a cover slip (Sigma). Cells were visualized in an epifluorescence microscope Olympus IX81. BrdU+ nuclei were counted and ratio of DAPI to BrdU labeling was compared among groups. Data were analyzed using analyzing tools of ImageJ software and shown as mean ± SEM, with *p* < 0.01 considered significant.

### Cultured microglia treated with masitinib

Microglial cells were plated in a 24-multiwell dish during 24 h and floating fat was removed. Masitinib treatment was started at that time and was repeated every 48 h chronically with each media change. Three doses of masitinib diluted in DMSO were tested, 0.1, 0.5, and 1.0 μM. Vehicle cells were treated with the same amount of DMSO as a control. Microphotographs were taken using a phase contrast microscope equipped with a Canon HD camera. Cells were treated during 15 days until cell transformation into monolayers of aberrant astrocyte cells. Quantitative analysis of the cell number every 48 h was assessed using the “cell counter” of the ImageJ Software. The number of cells in the masitinib-treated plates was compared to vehicle-treated ones. Data are shown as mean ± SEM, with *p* < 0.01 considered significant.

### Cell cultures from symptomatic SOD1^G93A^ masitinib-treated rats

After 20 days of treatment with 30 mg/kg/day of masitinib, spinal cords were cultured in p35 dishes (three different treated animals were cultured as described previously). Spinal cords from vehicle-treated animals were cultured as controls. After 24 h, floating fat was removed and pictures were taken every 48 h after every change of culture media, using a phase contrast microscope and a Canon HD camera. Pictures were taken during 10 days. Cells were counted using the tool “cell counter” from ImageJ Software. Data is shown as the number of microglia cells/mm^2^ in the masitinib-treated rats and compared to vehicle. Kruskal-Wallis analysis was used among groups. Data are shown as mean ± SEM, with *p* < 0.01 considered significant.

### Real-time PCR analysis in microglia cell cultures

Three different end-stage symptomatic rat spinal cords were cultured to obtained microglia as described previously [5]. Cells were plated in p60 dishes during 5 days and treated during 72 h with different doses of masitinib (0.5–1 μM) in DMEM-10 % FBS. An estimated 200,000 cells were processed for each mRNA extraction using RNeasy Micro kit (QIAGEN) according to the manufacturer’s instructions. mRNA yields were measured on Nanodrop device (Thermo Scientific) and cDNA were obtained from 0.5 μg of RNA (−80 °C), 4 μL of iScript reverse transcription Supermix for RT-qPCR (BIORAD, −20 °C) in a final volume of 20 μL filled with nuclease free water. The Thermo cycler was set as follows: priming 5 min at 25 °C followed by 30 min at 42 °C for reverse transcription and 5′ at 85 °C for RT inactivation. RT-qPCR was performed on reverse transcribed cDNA using SsoAdvanced™ Universal SYBR^®^ Green Supermix (BIO-RAD) on a CFX96 Touch™ real-time PCR detection system. For each well, 5 μL of diluted DNA was added to 20 μL of mix (containing 1 μL of each primer, 12.5 μL of SsoAdvanced™ Universal SYBR^®^ Green Supermix, 5.5 μL of nuclease free water). Each sample was run in duplicate. The cycling parameters were as follows: 30 s at 95 °C then 40 cycles at 95 °C for 10 and 30 s at 60 °C. Cq values were obtained for every cycle. Primers were designed on NCBI Primer-BLAST following the best guidelines to exclude genomic DNA amplification. The analysis was done using BioRad CFX manager 3.1 with a threshold set at 650 RFU corresponding to the amplification curves linear portion. Variations between samples were normalized using two housekeeping genes PGK1 and HPRT. All primers were validated for specificity and efficiency. Primers were designed on PrimerBlast. All primers were validated with differentiated bone marrow-derived rat macrophages (BMDM) in vitro and selected for specificity and quantitativity before being tested on primary microglia from SOD1^G93A^ rats (only primers achieving quantitatively up to a dilution factor of 500 were kept). The following primers were used: monocyte chemoattractant protein-1 (**MCP**-**1**) forward 5′-TGT CTC AGC CAG ATG CAG TTA AT-3′; reverse 5′-TCC AGC CGA CTC ATT GGG AT-3′; Interleukin-6 (**IL**-**6**) forward 5′-TTC TCT CCG CAA GAG ACT TCC-3′; reverse 5′-TCT CCT CTC CGG ACT TGT GAA-3′; tumor necrosis factor alpha (**TNFα**) forward 5′-ATC CGA GAT GTG GAA CTG GC-3′; reverse 5′-TGG GAA CTT CTC CTC CTT GTT G-3′; inducible nitric oxide synthase (**iNOS**) forward 5′-AGC CTA GTC AAC TAC AAG CCC C-3′; reverse 5′-CAT CCT GTG TTG TTG GGC TG-3′; interleukin-1 beta (**IL**-**1β**) forward 5′-TAG CAG CTT TCG ACA GTG AGG-3′; reverse 5′-CTC CAC GGG CAA GAC ATA GG-3′; cyclooxygenase-2 (**Cox2**) forward 5′-TGT ACT ACG CCT GAG TTT CTG AC-3′; reverse 5′-GGG ATC CGG GAT GAA CTC TC-3′; ionized calcium-binding adaptor molecule 1 (**Iba1**) forward 5′-CAA GGA TTT GCA GGG AGG AAA A-3′; reverse 5′-TTG AAG GCC TCC AGT TTG GAC-3′; transcription factor Spi-1/PU.1 **(PU.1)** forward 5′-GGA GAC AGG CAG CAA GAA GAA G-3′; reverse 5′-CCT TCA TGT CTC CGC TAC GC-3′; hypoxanthine-guanine phosphoribosyl transferase (**HPRT**) forward 5′-GTC ATG TCG ACC CTC AGT CC-3′; reverse 5′-GCA AGT CTT TCA GTC CTG TCC-3′; phosphoglycerate kinase 1 (**PGK1**) forward 5′-GTC GTG ATG AGG GTG GAC TT-3′; reverse 5′-AAC CGA CTT GGC TCC ATT GT-3′.

### Kinase inhibition assay

CSF-1R kinase domain (AA 538–972) was expressed as a *N*-terminus 6HN-tagged protein in Sf21 cells using the BacPAK6 baculovirus expression system (Clontech, Mountain View, CA 94043, USA) and purified by Ni^2+^ affinity chromatography. The analysis of the effect of masitinib on CSF-1R kinase activity was assessed with the *HTRF*^®^*KinEASE assay* (Cisbio International, Bagnols-sur-Cèze, France) using a biotinylated poly(Glu_4_Tyr) peptide (1 μM) as substrate. Kinase assays were performed at an ATP concentration of 100 μM (CSF-1R Km_ATP_ = 52 μM) in kinase buffer (50 mM HEPES pH 7.5, 5 mM MgCl_2_, 1 mM MnCl_2_, 1 mM DTT, 0.01 % Brij-35) for 30 min at room temperature in the presence of various masitinib concentrations (0 to 10 μM). Reactions were stopped by addition of EDTA, and samples were incubated for 1 h with an anti-phospho peptide-Eu^3+^ antibody (emission 620 nm) and streptavidin XL-665 (emission 665 nm) according to manufacturer’s instructions. After incubation, the obtained signal is proportional to the concentration of phosphorylated peptide in the sample. All measurements were performed on a BMG Labtech Pherastar FS apparatus. Results are expressed in delta fluorescence (DF) unit defined as follow DF % = [(ratio − ratio blank) / (ratio blank)] × 100, where ratio = (665 nm/620 nm) × 10^4^. Each experiment was performed in duplicate and repeated three times.

### Statistics analysis and survival curves

Survival curves were compared by Kaplan-Meier analysis with the log-rank test using PAST3 software. Quantitative data were expressed as mean ± SEM and Student’s *t* test or ANOVA followed by Scheffé post hoc comparison if necessary were used for statistical analysis, with *p* < 0.01 considered significant.

## Results

### Masitinib prevents M-CSF-induced proliferation in cultured microglia

**Fig. 1:**
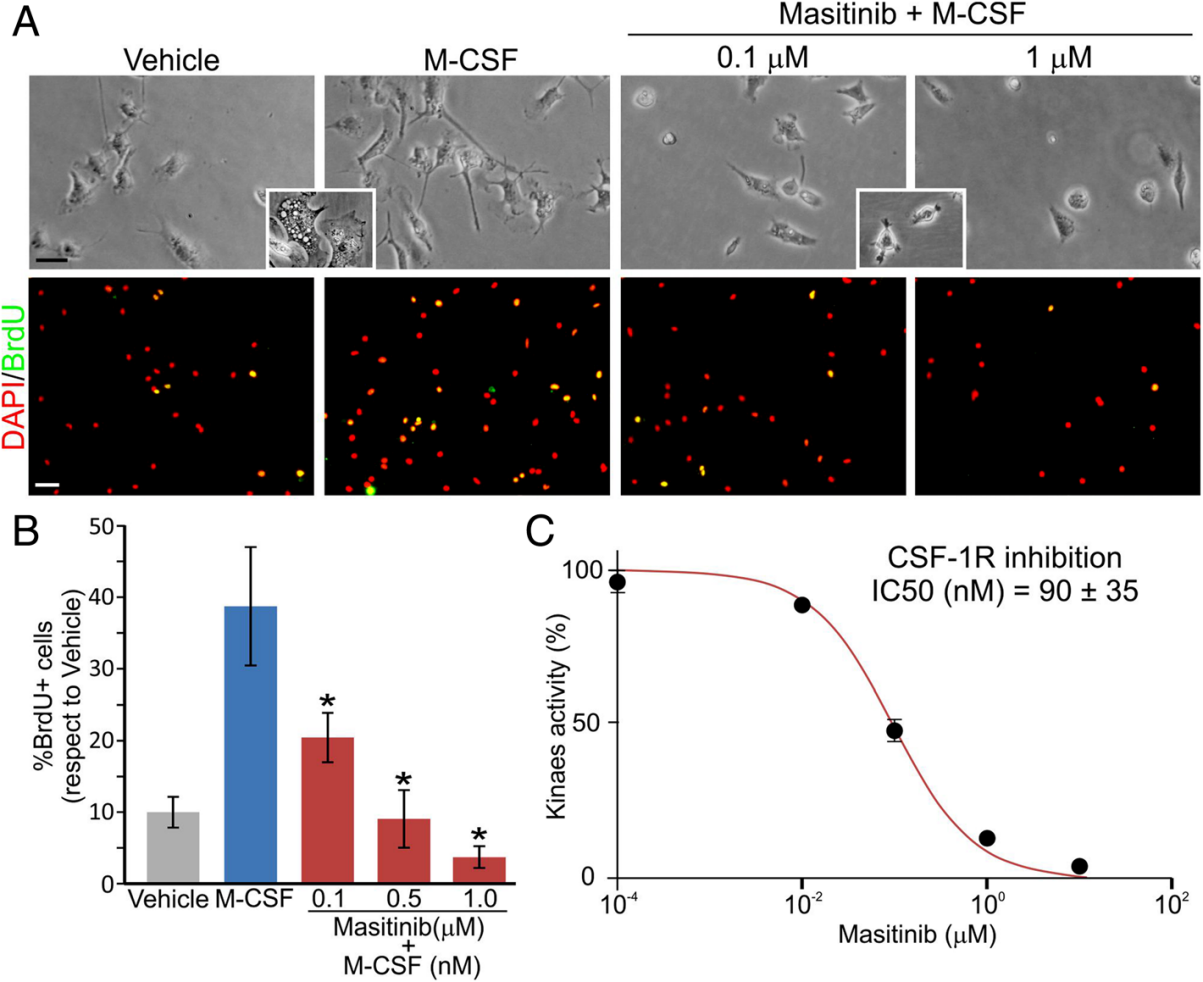
Masitinib prevented microglia proliferation by inhibiting CSF-1R. **a** Microglia cultured from symptomatic SOD1^G93A^ rat spinal cord in low FBS conditions (0.5 %) adding 30 ng/mL of M-CSF with indicated masitinib concentrations. The *insets* show the hypertrophic vacuolated cells in vehicle and M-CSF conditions and the small rounded cells after masitinib treatment (*scale bars*: contrast 20 μm and DAPI/BrdU 50 μm). **b** The *graph* shows the quantitative BrdU analysis where positive cells were counted and expressed as percentage of total cells stained with DAPI. M-CSF treatment produced a significant increase in microglial proliferation that was blocked by masitinib. **c** Kinase inhibition assay showing that masitinib inhibited CSF-1R with an IC_50_ = 90 ± 35 nM. All data are expressed as mean ± SEM *p < 0.01

To determine the effect of tyrosine kinase inhibition with masitinib, we used microglia isolated from the primary spinal cord cultures of symptomatic SOD1^G93A^ rats before their transformation into astrocyte-like cells [5]. Microglia appeared as hypertrophic phagocytic cells that actively proliferate in the presence of fetal bovine serum (FBS) or M-CSF (Fig. 1a). Treating cell cultures using pharmacological concentrations of masitinib (0.1–1 μM) dose-dependently abrogated the morphology of hypertrophic phagocytic microglia and also inhibited M-CSF-induced proliferation as measured by BrdU uptake (Fig. 1b). In accordance, masitinib potently inhibited the kinase activity of recombinant CSF-1R with an IC_50_ of 90 ± 35 nM (Fig. 1c), a concentration that is reachable in vivo.

### Masitinib prevents SOD1^G93A^ microglia cells inflammatory profile

**Fig. 2:**
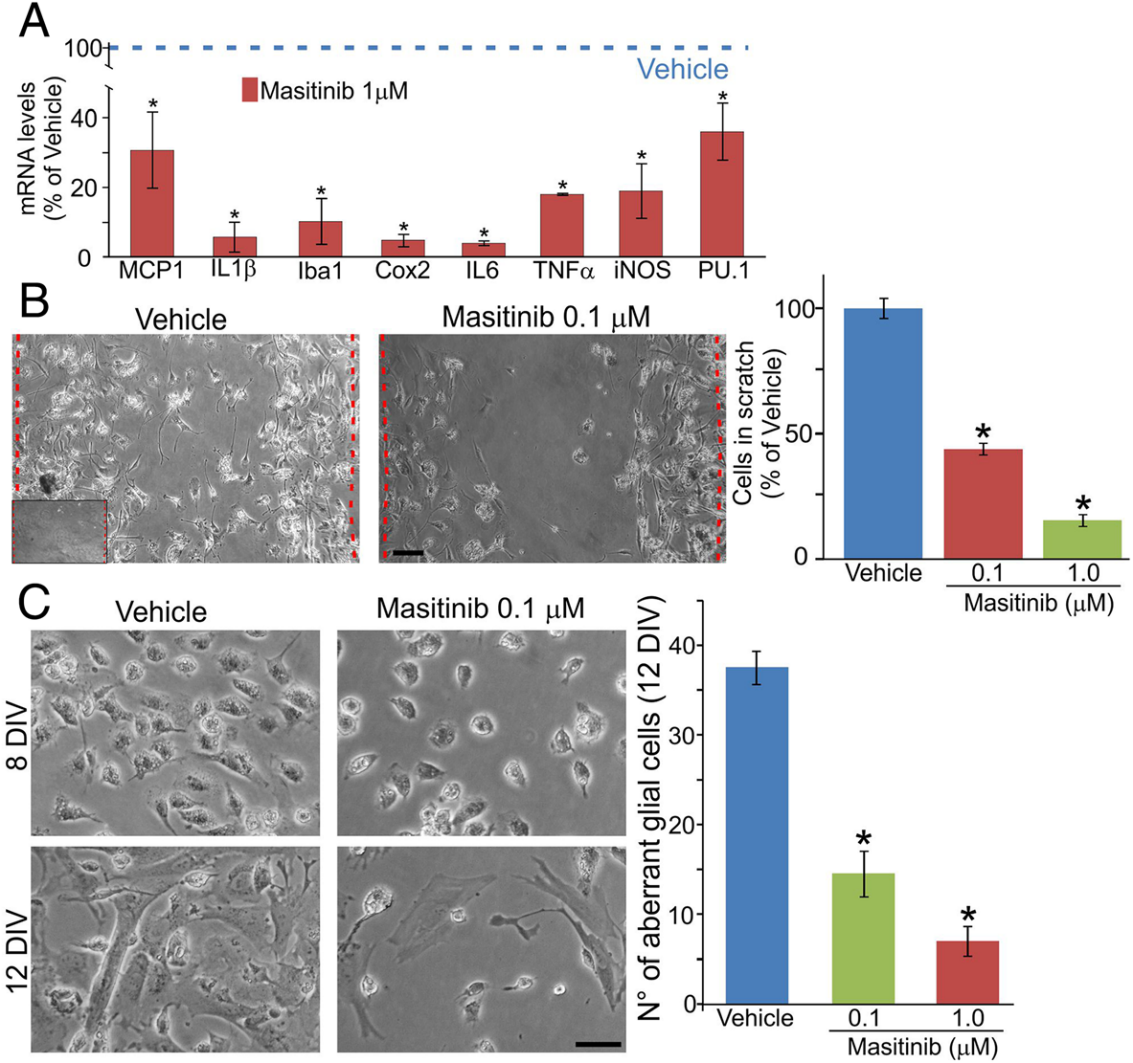
Masitinib inhibited microglia proinflammatory phenotype and prevented microglia migration and transformation into aberrant glial cells. **a** Real-time PCR analysis showed that the treatment with pharmacological concentration (1 μM) of masitinib during 72 h is sufficient to significantly reduce the expression of several genes involved in inflammatory processes. **b** A confluent monolayer was scratched to determine the migratory capacity of aberrant microglia. After 24 h, cells located between the *dashed lines* were counted. Vehicle-treated microglia covered most of the scratch after 24 h, while masitinib-treated cells showed significantly less migratory capacity. The *inset* shows the open space in the monolayer immediately after making the scratch (*scale bar* 20 μm). The graph to the *right* shows the quantitative analysis of migration. **c** Masitinib prevented microglia transformation into aberrant glial cells in a dose-dependent manner when compared with vehicle-treated cultures. Note how after 12 days in vitro, microglia transition to a flat astrocyte-like cell that reach confluence. Masitinib significantly prevented this transformation and few microglia cells transitioned into aberrant glial cells (*scale bar* 10 μm). The graph to the *right* represents the quantitative analysis showing the number of aberrant glial cells after 12 days in vitro (12DIV). All data are expressed as mean ± SEM *p < 0.01

As predicted by the hypertrophic and phagocytic morphology, primary cultured microglia from symptomatic SOD1^G93A^ spinal cords displayed a robust transcriptional activity for inflammatory genes. After exposure to masitinib during 72 h, the transcription of several genes highly involved in neuroinflammation decreased more than 50 %. In particular, relevant inflammatory transcripts such as IL1β, IL6, Iba1, and Cox2 were downregulated by approximately 80 % (Fig. 2a). In addition, Fig. 2b shows that masitinib inhibited by more than 50 %, the ability of microglia to migrate across a scratch made in the culture dish in low FBS conditions.

Previously, we have reported that hypertrophic microglia from ALS rats follow a phenotypic transition after 12–15 days in culture turning to flat, astrocyte-like cells characterized by being highly toxic for cultured motor neurons [4, 5]. Figure 2c shows that masitinib (0.1–1 μM) potently prevented this phenotypic transformation by more than 50 %, thus preventing the emergence of the aberrant glial cell phenotype in culture.

### Post-paralysis masitinib treatment reduces the number of aberrant glial cells and neuroinflammation

**Fig. 3:**
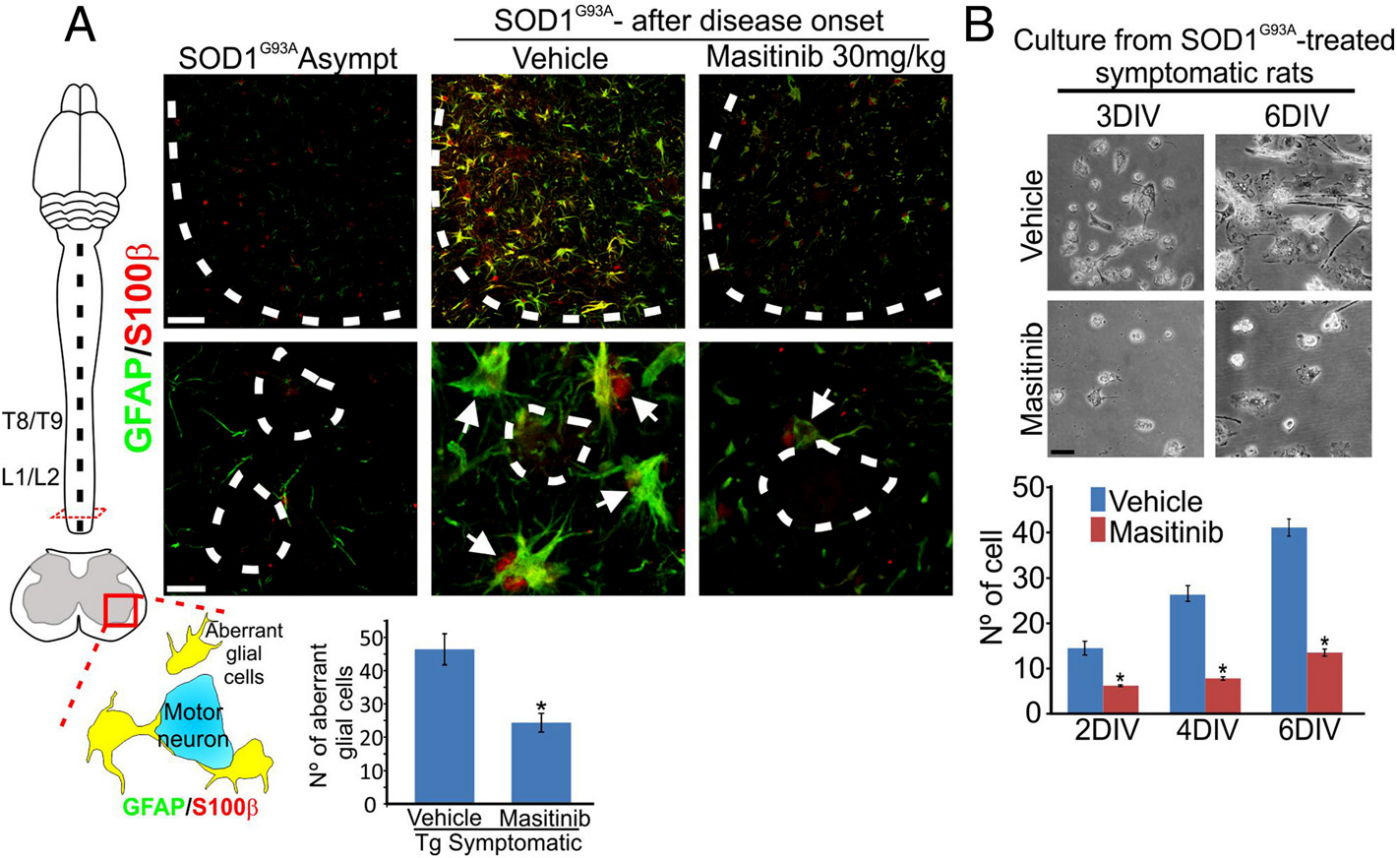
Masitinib treatment reduced the number of aberrant glial cells in the degenerating spinal cord. **a** Aberrant glial cells expressing GFAP (*green*) and S100β (*red*) in the ventral horn of the spinal cord. *Dotted white lines* mark the border between white and grey matter in the upper images and outline motor neurons in high magnification panels. In the vehicle-treated rats aberrant glial cells (*white arrows*) surround motor neurons as compared with asymptomatic (Tg Asympt) where glial cells express low S100β. Masitinib (30 mg/kg/day) prevented the appearance of aberrant glial cells in the degenerating spinal cord after 20 days treatment. The scheme represents the level of spinal cord segments and Rexed laminae VII and IX where aberrant glial cells were counted (*scale bars* 50 μm in low magnification and 10 μm in high magnification). **b** Spinal cord culture from masitinib-treated rats compared with vehicle-treated rats. Relatively few cells were obtained from the degenerating spinal cord after masitinib treatment when compared with vehicle-treated animals (*scale bar* 15 μm). All data are expressed as mean ± SEM *p < 0.01

We then explored whether chronic treatment with masitinib could reduce the number of aberrant glial cells in the degenerating spinal cord, which were identifiable as large GFAP/S100β-positive cells located around motor neurons as described previously [4]. Rats were orally treated with masitinib (30 mg/kg/day), starting right after paralysis onset and during the next 20 days, corresponding to the average post-paralysis survival in untreated rats (Fig. 5). Only rats that initiated paralysis in the hind limbs were used in the experiments in order to reduce experimental variables. As compared with rats treated with vehicle, masitinib significantly reduced the number of aberrant glial cells in the lumbar spinal cord by 40 % (Fig. 3a).

In accordance, masitinib treatment to symptomatic rats prevented the isolation and subsequent proliferation of microglia in primary cell cultures of the degenerating spinal cord (Fig. 3b). This sharply contrasted with a large number of hypertrophic and phagocytic cells obtained in cultures from vehicle-treated rats, indicating that masitinib treatment reduces inflammatory and proliferative potential of endogenous glial cells.

**Fig. 4:**
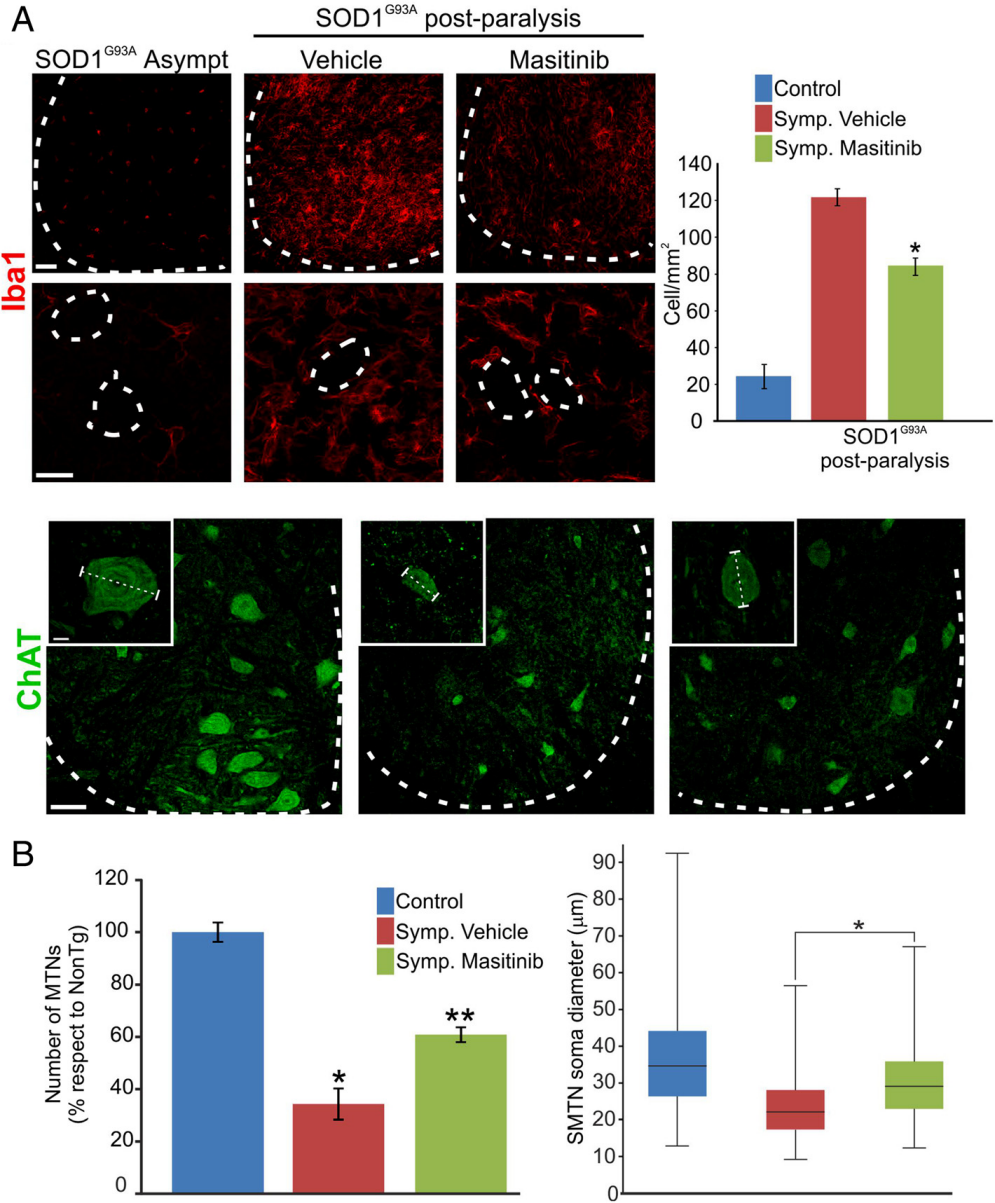
Masitinib ameliorates microgliosis and motor neuron pathology. **a** Microglia marker Iba1 confocal images. Note the significant reduction of microglial cells in masitinib-treated rat spinal cords when compared with vehicle-treated ones. High magnification panels show a significant reduction in the number of microglial cells that surround motor neurons after masitinib chronic treatment (*scale bars* 50 μm low magnification and 20 μm high magnification). **b** Confocal image of ChAT in the lumbar spinal cord (*dotted line*) indicates the border between white and grey matter (*scale bar* 50 μm). The *graph* below to the *left* represents the quantitative analysis showing the number of motor neurons in the ventral horn in each condition. The *graph* to the *right* represents the quantitation of the motor neuron soma diameter showing the decreased diameter of surviving motor neurons in vehicle-treated rats and the protective effect of masitinib (*insets* in a) (*scale bar* 10 μm). All data are expressed as mean ± SEM *p < 0.01, **p < 0.01

Post-paralysis masitinib treatment also significantly reduced microgliosis as assessed by the number of cells expressing Iba1+, CD206+, or CD68+ cells in the ventral horn of the lumbar spinal cord, when compared with vehicle-treated animals (Fig. 4a, see Additional file 1: Figure S1A). Remarkably, there was a reduction of hypertrophic Iba1+ microglia cells surrounding motor neurons at thoracic and cervical levels of the degenerating spinal cords, suggesting that masitinib may prevented the spread of neuroinflammation along the neuraxis (see Additional file 1: Figure S1B).

### Masitinib ameliorates motor neuron pathology

Because motor neuron death is the main pathological feature of symptomatic rodent models and human ALS, we used the same experimental setting to determine if motor neuron pathology was influenced by masitinib treatment. As shown in Fig. 4b, in vehicle-treated SOD1^G93A^ rat, the number of ventral horn ChAT+ motor neurons decreased by 60 % when measured 20 days after paralysis onset. In comparison, masitinib significantly reduced motor neuron loss to 40 % when administered after paralysis onset. We next analyzed the effect of masitinib treatment on the motor neuron soma diameter in surviving motor neurons. When compared with non-transgenic animals (soma diameter 35.8 ± 8.7 μm), there was a significant reduction in the soma diameter of end-stage symptomatic rats (23.4 ± 5.8 μm). This neuron atrophy was significantly prevented by masitinib treatment (30 ± 10.3 μm) (Fig. 4b).

### Masitinib prolongs post-paralysis survival in SOD1^G93A^ rats

**Fig. 5:**
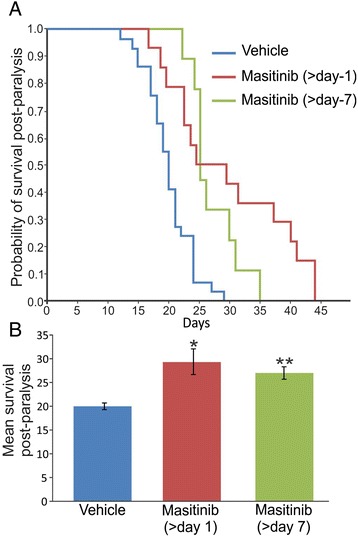
Masitinib treatment after paralysis onset increased survival of SOD1^G93A^ transgenic rats. **a** Kaplan-Meier survival curves from masitinib-treated and vehicle-treated SOD1^G93A^ rats. SOD1 ^G93A^ transgenic rats were treated with masitinib (30 mg/kg/day) or vehicle (water, *n* = 29, *blue line*) immediately after observation of paralysis onset of one limb (day 1; *n* = 14, *red line*) or starting 7 days after paralysis onset (day 7, *n* = 9, *green line*). There was a statistically significant difference in the probability of survival for both masitinib-treated groups when compared with vehicle-treated group, according to the log-rank test of the Kaplan-Meier analysis (*p* < 0.0006 for masitinib—gait onset vs. vehicle and *p* < 0.00025 for masitinib—7 days onset vs. vehicle). **b** The *graph* shows the mean survival of the three different groups. All data are expressed as mean ± SEM. *p* < 0.01 was considered significant

Next, we designed two randomized trials using masitinib to determine how the drug affected the survival of SOD1^G93A^ rats with hind limb onset. As shown in Table 1, our rat colony develops disease with a delayed onset (187 ± 15 days for vehicle rats) if compared with that originally described by Howland et al. [15]. In our colony, the post-paralysis survival with hind limb onset has been highly reproducible in non-treated animals (20 ± 3.8 days, Fig. 5). Table 1 shows that there were no significant differences in the pre-treatment parameters analyzed such as age and weight between treated and non-treated animal groups (see Additional file 1: Figure S2C, D).

**Table 1:** Characteristics of SOD1^G93A^ rats used in the post-paralysis masitinib trial

	Vehicle	Masitinib (>day 1)	Masitinib (>day 7)
Age at onset (days)	187 ± 15	182 ± 25	198 ± 14
Weight at onset (g)	315 ± 56	306 ± 16	323 ± 71
Weight at end-stage (g)	235 ± 13	214 ± 50	211 ± 50
Survival range (days)	174–234	177–249	201–246

The table shows characteristics of the consolidated studies for rats treated with vehicle, masitinib starting 1 (>day 1) and 7 (>day 7) days after paralysis onset. Age and weight values are expressed as mean ± SD. Survival range indicates the age of rats at the time animals reached end-stage of paralysis

Masitinib (30 mg/kg/day) was administrated immediately upon abnormal gait onset (day 1) or after day 7. The effect of masitinib treatment was compared against age, weight, and gender-matched control groups treated with vehicle (Fig. 5a, b, see Additional file 1: Figure S2A–D). Without masitinib, the ALS rats died within 3 weeks of paralysis onset (Fig. 5a). Rats in the masitinib day 1 cohort (*n* = 14) had a statistically significant (*p* < 0.0006) difference in the probability of survival when compared with vehicle-treated animals (Kaplan-Meier curves, Fig. 5a). Remarkably, animals in the masitinib day 7 cohort (*n* = 9) also had a statistically significant (*p* < 0.0001) difference in the probability of survival with respect to controls.

Comparing each cohort’s mean survival time also evidenced a survival benefit for masitinib. The mean post-paralysis survival time was respectively 30 ± 8 days and 27 ± 4.3 days for day 1 and day 7 masitinib cohorts, which were both significantly longer than the 20 ± 3.8 days observed for vehicle-treated rats (Fig. 5b, *p* < 0.0016 and 0.0003, respectively). The protective effect of masitinib was equally observed in both female and male rats (see Additional file 1: Figure S2A, B).

## Discussion

Tyrosine kinase inhibitors are a well-established class of drugs typically used to suppress or decrease cancer cell proliferation and modulate the associated tumor microenvironment [17, 18]. Here, we show evidence that the tyrosine kinase inhibitor masitinib therapeutically modulates the neuroinflammation associated with the ALS progression. Masitinib reduced microgliosis and the subsequent emergence of aberrant glial cells in the degenerating spinal cord, which is consistent with a potent effect in microglial cell cultures, downregulating proliferation, migration, and inflammatory transcriptional profile. Remarkably, treatment of already paralytic rats with masitinib resulted in an unprecedented increase (~40 %) in post-paralysis survival in both genders. While other drugs modulating glial cell inflammation can prolong survival in ALS models [19], our study is the first one showing a protective effect when the drug is delivered post-paralysis. Such a therapeutic approach is appealing in the clinical setting of ALS where drug treatment is initiated only after overt motor symptoms.

Tyrosine kinase inhibition with masitinib sharply decreased the number microglia cells expressing Iba1, CD206, and CD68 and the appearance of aberrant glial cells in the lumbar spinal cord, thus supporting the concept that preventing the emergence of aberrant glial cells moderates the accelerated paralysis progression characteristic of SOD1^G93A^ rats. Our results also anticipate that tyrosine kinase inhibition could be also protective in other ALS models involving overt glial activation [20, 21]. Indeed, the SOD1^G93A^ rat appears as a useful model to study drugs with post-paralysis effects through the modulation of CNS neuroinflammation, contrasting with SOD1^G93A^ mice where distal axonopathy appears as a more important determinant of post-paralysis survival [15, 22].

The inhibition of receptor and non-receptor tyrosine kinases controlling inflammation and exaggerated glial cell activation appears as the most plausible mechanism of action of masitinib. Compared to previous studies in ALS murine models based on ablation of proliferating microglial cells [23–25], treatment with masitinib does not eliminate the proliferating microglia. Rather, it more likely modulates the proliferation and inflammatory signaling underlying the emergence of aberrant glial cells, representing a new pharmacological approach to control detrimental neuroinflammation.

In particular, we found that masitinib inhibits purified recombinant CSF-1R kinase activity at nanomolar concentrations and reduces M-CSF-induced microglia proliferation and migration ability in vitro, suggesting that it regulates a key inflammatory pathway, thus promoting microglia expansion and invasive behavior. Previous reports have shown that activation of CSF-1R by the agonist M-CSF or interleukin 34 potently regulates macrophage/microglia number and inflammatory phenotype in animal models [26, 27]. Recent reports have shown that motor neurons express M-CSF upon damage causing the expansion of surrounding spinal microglial cells [9, 28], thus prompting a pathogenic pathway where motor neuron pathology exacerbates deleterious microgliosis. In accordance, M-CSF levels are elevated in ALS patients as well as in ALS mouse models and may represent a key pathway exacerbating microgliosis and ALS progression [29–31]. Moreover, a recent report has shown that CSF-1R blockade with the drug GW2580 administered to ALS mice several weeks before paralysis onset decreased both microgliosis and slowed disease progression [32].

Although masitinib is a relatively selective kinase inhibitor [33] for CSF-1R, it also targets a few other tyrosine kinases such as PDGF-R, c-Kit, Lyn, and Fyn [10, 11], whose activation may also be associated with the modulation of the neurodegenerative microenvironment. A number of tyrosine kinase inhibitors targeting specific receptors have been approved over the last 5 years for many different types of cancer. However, there are few reports of central nervous system adverse effects or direct neuronal damage. The effect of masitinib, as well as other tyrosine kinase inhibitors, is not selective for a specific cell type, because it blocks several kinases expressed in many cell types. However, masitinib is one of the most selective kinase inhibitors currently in development and as such, potentially exerts a low toxicity profile [11]. In addition, the effects of inhibition of kinase targeted by masitinib are more involved in cell proliferation (c-Kit, PDGF-R, and MCSF/CSF-1R) and immune activation (Lyn, Fyn) than apoptosis. Thus, post-mitotic cells and particularly neurons and resting astrocytes are generally less vulnerable to pharmacological tyrosine kinase inhibition by masitinib as reported previously [13, 34]. Furthermore, inhibition of the non-receptor c-Abl kinase by imatinib has been shown to prevent astrocyte-induced motor neuron death in cell cultures [34], further suggesting an alternative neuroprotective pathway unrelated to neuroinflammation. Such a unique combination of molecular effects could explain our results showing the potent effect of masitinib downregulating the expression of inflammatory mediators, characteristic of deleterious aberrant glial cell phenotype. Thus, further work will be required to decipher the precise tyrosine kinases deregulated in ALS and their pharmacological targeting.

The present study does not establish whether masitinib targets inflammatory cells outside the CNS also known to influence motor neuron degeneration [35, 36]. In particular, masitinib may target the peripheral monocyte/macrophage system, which appears affected in ALS animal models. Immunological downregulation of Ly6C^hi^ monocytes that infiltrate the degenerative spinal cord attenuates motor neuron loss and delays disease progression in mutant SOD1 mice [37]. Masitinib could also target macrophages that infiltrate and promote degeneration of peripheral motor axons [38]. Moreover, macrophage activation and microgliosis are known to be influenced by mast cells located inside or outside the blood-brain barrier [39]. Because masitinib potently prevents mast cell differentiation and activation [10], it could also indirectly regulate neuroinflammation by targeting mast cells through the inhibition of c-Kit, Lyn, and Fyn. Future studies in patients and animal models are needed to determine alternative mechanisms of action of tyrosine kinase inhibitors and masitinib in ALS.

There are currently no effective treatments for ALS. Riluzole, an anti-glutamatergic drug, is the sole authorized product used in ALS, providing a modest improvement in survival (~3 months) [40]. Over the past 10 years, a number of drugs were identified as providing survival benefits in rodent preclinical trials [19]. However, none of them proved to be clinically better than riluzole in ALS patients. Such incongruence could be explained, at least in part, by the fact that most animal studies that were translated to clinical trials have been started before paralysis onset. Therefore, tyrosine kinase inhibition with masitinib appears unique among other ALS-developmental drugs because it exerts neuroprotection when administrated post-paralysis. A randomized phase III clinical trial testing the effect of masitinib in ALS patients is currently running (Clinicaltrial.gov NCT02588677).

## Conclusions

The present study shows that tyrosine kinase inhibition with masitinib is capable of controlling microgliosis, neuroinflammation, and the emergence/expansion of aberrant glial cells in SOD1^G93A^ rats. Remarkably, masitinib significantly prolonged survival when delivered after paralysis onset, an unprecedented effect in preclinical models of ALS, and therefore appears well-suited for treating ALS where drug treatment is initiated only after diagnosis based on overt motor symptoms.

## Abbreviations

ALS, amyotrophic lateral sclerosis; BrdU, bromodeoxyuridine; ChAT, choline acetyltransferase; c-Kit, stem cell growth factor receptor (SCFR); CNS, central nervous system; Cox2, cyclooxygenase-2; CSF-1R, colony-stimulating factor 1 receptor; FBS, fetal bovine serum; FLT3, fms-related tyrosine kinase 3; Iba1, ionized calcium binding adaptor molecule 1; IL1β, interleukin 1 beta; IL6, interleukin 6; M-CSF, macrophages colony-stimulating factor; PDGF-R, platelet-derived growth factor receptor; SOD1, superoxide dismutase 1

## Additional file

Additional file 1:
**Figures S1.** Masitinib inhibited microgliosis along the degenerating spinal cord. **Figure S2.** Masitinib treatment after paralysis onset increased survival of SOD1^G93A^ female and male rats. (DOCX 700 kb)
