# Supplementary material for: Post-paralysis tyrosine kinase inhibition with masitinib abrogates neuroinflammation and slows disease progression in inherited amyotrophic lateral sclerosis
Source: J Neuroinflammation. 2016 Jul 11;13:177. doi: 10.1186/s12974-016-0620-9 (PMC4940876; doi:10.1186/s12974-016-0620-9)
Supplement: Additional file 1: — Figures S1. Masitinib inhibited microgliosis along the degenerating spinal cord. Figure S2. Masitinib treatment after paralysis onset increased survival of SOD1G93A female and male rats. (DOCX 700 kb) [file 12974_2016_620_MOESM1_ESM.docx]

**Additional file 1**

**Figure S1. Masitinib inhibited microgliosis along the degenerating spinal cord. A)** Confocal images showing two microglia markers, CD206 and CD68 in the degenerating spinal cord of masitinib-treated animals in comparison with vehicle-treated animals and transgenic asymptomatic rats. (Scale bars: 50 μm). **B)** Histological analysis of disease spreading along the neuraxis. The schemes at left represent the level of spinal cord segments where images where obtained; dotted lines indicate the border between white and grey matter in low magnification or outline the motor neuron somas in high magnification images. Masitinib also reduced the levels of microglia cells in the thoracic and cervical spinal cord when compared with control animals. (Scale bar: 50 μm low magnification panels and 20 μm high magnification panels). All results are presented as mean ± SEM; *p < 0.01 was considered significant.

**Figure S2.** **Masitinib treatment after paralysis onset increased survival of SOD1^G93A^ female and male rats (A)** Kaplan-Meier survival curves from female masitinib-treated and vehicle-treated rats. Red line represents survival in a group of 7 female rats that were treated with masitinib (30 mg/kg/day) immediately after observation of disease onset (gait). Green line represents survival in a group of 4 female rats that were treated with masitinib (30 mg/kg/day) from 7 days post observation of disease onset (gait). Blue line represents survival after paralysis onset in a group of 14 female vehicle-treated control rats. Both masitinib treatment groups showed a significant difference in the probability of survival when compared with vehicle-treated rats (p<0.025 and p<0.0016, respectively). The graph below shows the quantitative analysis of the mean survival in aforementioned treatment groups (*p<0.041; **p<0.003). **(B)** Kaplan-Meier survival curves from male masitinib-treated and vehicle-treated rats. Red line represents survival in a group of 7 male rats that were treated with masitinib (30 mg/kg/day) immediately after observation of disease onset (gait). Green line represents survival in a group of 5 male rats that were treated with masitinib (30 mg/kg/day) from 7 days post observation of disease onset (gait). Blue line represents survival after paralysis onset in a group of 15 male vehicle-treated control rats. Both masitinib treatment groups showed a significant difference in the probability of survival when compared with vehicle-treated rats (p<0.017 and p<0.016, respectively). The graph shows the quantitative analysis of the mean survival in aforementioned treatment groups (*p<0.04; **p<0.01). **(C)** Quantitative analysis of median age of animals at onset. There were no statistical significant differences among groups. **(D)** Quantitative analysis of the median weight of female and male SOD1^G93A^ rats at onset. There were no statistical significant differences among the analyzed groups. All results are presented as median ± SD *p < 0.01 was considered significant.

**Figure S1**

**
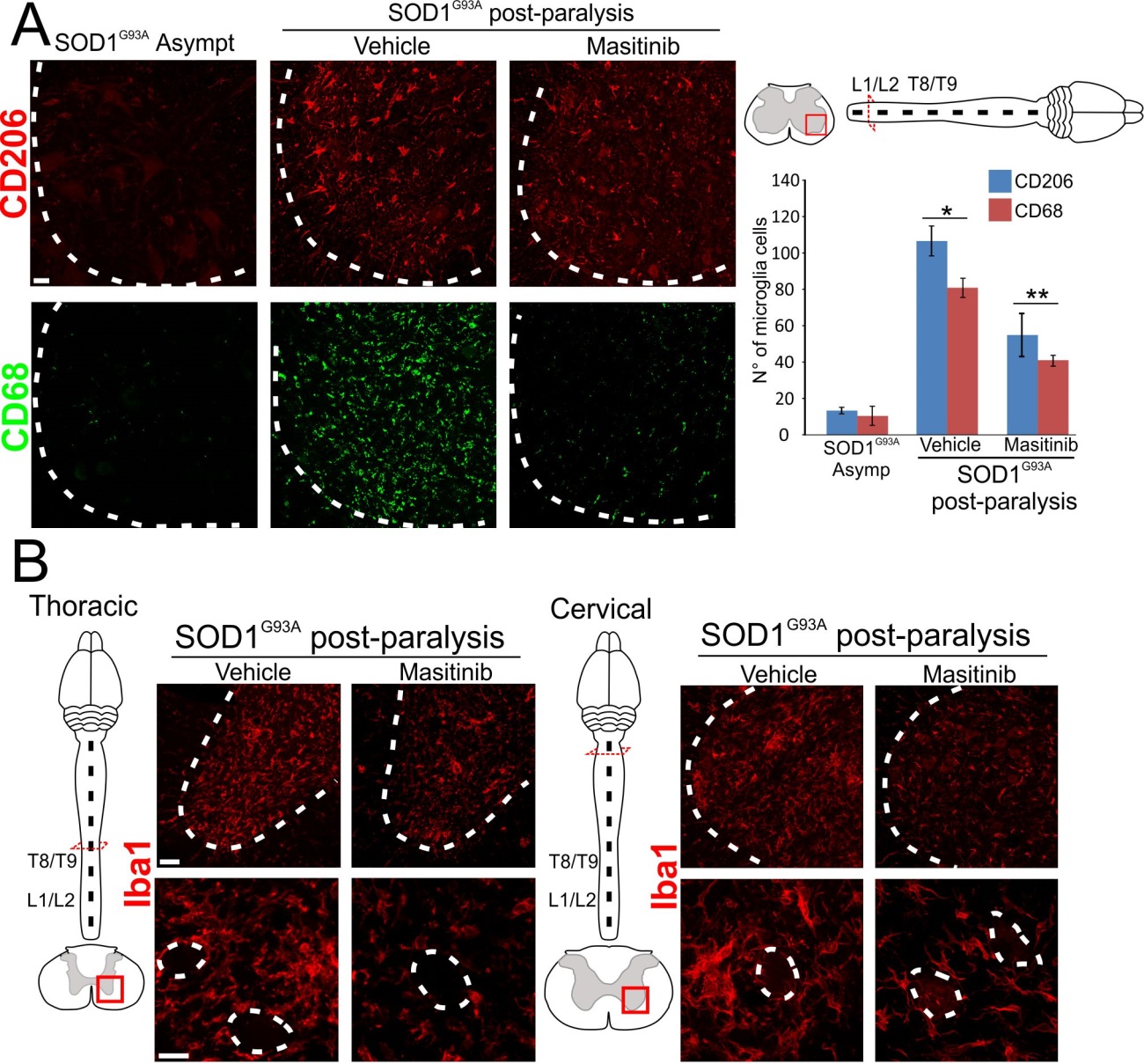
**

**Figure S2**

**
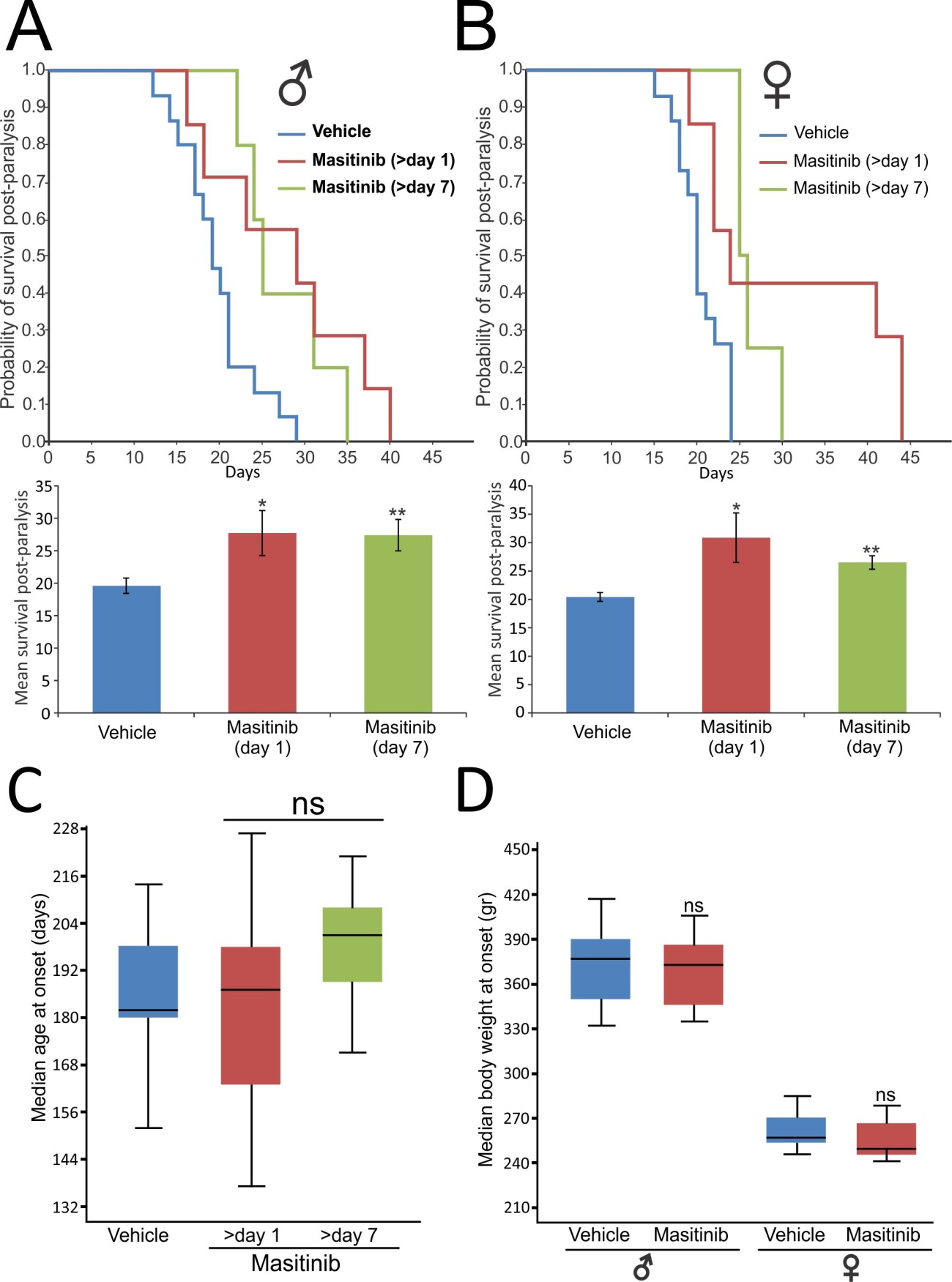
**
